# Intraoral Dissection of the Mimetic Muscles: Application to Dentistry and Oral Surgery

**DOI:** 10.7759/cureus.1939

**Published:** 2017-12-13

**Authors:** Joe Iwanaga, Koichi Watanabe, Jingo Kusukawa, Rod J Oskouian, R. Shane Tubbs

**Affiliations:** 1 Seattle Science Foundation; 2 Department of Anatomy, Kurume University School of Medicine; 3 Dental and Oral Medical Center, Kurume University School of Medicine; 4 Swedish Neuroscience Institute; 5 Neurosurgery, Seattle Science Foundation

**Keywords:** mimetic muscle, anatomy, cadaver, oral cavity

## Abstract

Mimetic muscles contract and pull the overlying skin toward the muscle’s bony attachment. Numerous books and articles have shown the mimetic muscles via cadaveric dissection. However, for dentistry and oral surgery, the mimetic muscles have not been detailed from intraoral dissection. Recently, several papers have addressed various mimetic muscles in relation to intraoral dissection. However, to our knowledge, there has been no overview of these muscles beneath the oral mucosa. Here, we review the literature concerning the mimetic muscles as revealed during intraoral dissection, create novel illustrations, and discuss the relationship of these muscles with general dentistry and oral surgery. The mimetic muscles, which constitute the surface of the oral mucosa, the relationship of the labial and buccal frenulum and mimetic muscles, the relationship of the mucogingival junction and mimetic muscles, and other surgical procedures are discussed. A better understanding of the mimetic muscles from an intraoral perspective is important for those performing oral surgery and dentistry.

## Introduction and background

The mimetic muscles originate from the viscerocranium and attach to the overlying skin. Numerous anatomy books and articles containing drawings and photographs of the mimetic muscles have been published over the years and many of them have depicted these muscles in clear detail [1-2]. From a clinical perspective, many different surgical procedures have been developed, such as facelifts and cleft lip repair, and these necessitate knowledge of the mimetic muscles (origin, insertion, and function) [3-4]. Unfortunately, in regard to dentistry and oral surgery, the mimetic muscles have seldom been depicted in the context of intraoral dissection, although various procedures for the oral mucosa have been developed. Recently, several papers have addressed specific mimetic muscles in relation to intraoral dissection [5-8]. However, to our knowledge, there has been no overview of these structures beneath the oral mucosa. Here, we review previous publications concerning the various mimetic muscles as revealed during intraoral dissection, create novel illustrations, and discuss the relationship of these muscles with general dentistry and oral surgery.

## Review

Mimetic muscles constitute the surface of the oral mucosa

**Figure 1:**
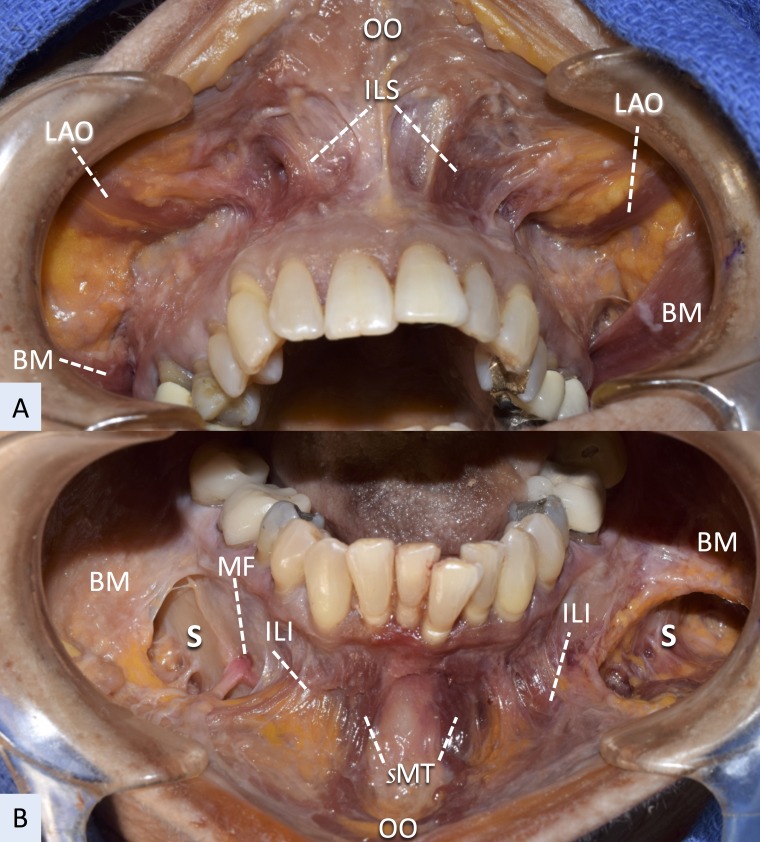
Intraoral dissection of the mimetic muscles using a fresh cadaver A: maxilla B: mandible BM; buccinator, ILI; incisivus labii inferioris, ILS; incisivus labii superioris, LAO; levator anguli oris, MF; mental foramen, OO; orbicularis oris, S; buccomandibular space, sMT; superior portion of the mentalis

The muscle fibers that extend vertically toward the orbicularis oris (OO) from the incisive fossa of the maxilla are called the incisivus labii superioris (ILS) or accessory skeletal heads of the OO [9]. The mentalis (MT) has two portions; the upper portion travels to the OO and the lower chin [8]. The lateral portion of the ILS and the incisivus labii inferioris (ILI) muscle join the OO obliquely and proceed to the corner of the mouth (Figure 1) [8,10].

**Figure 2:**
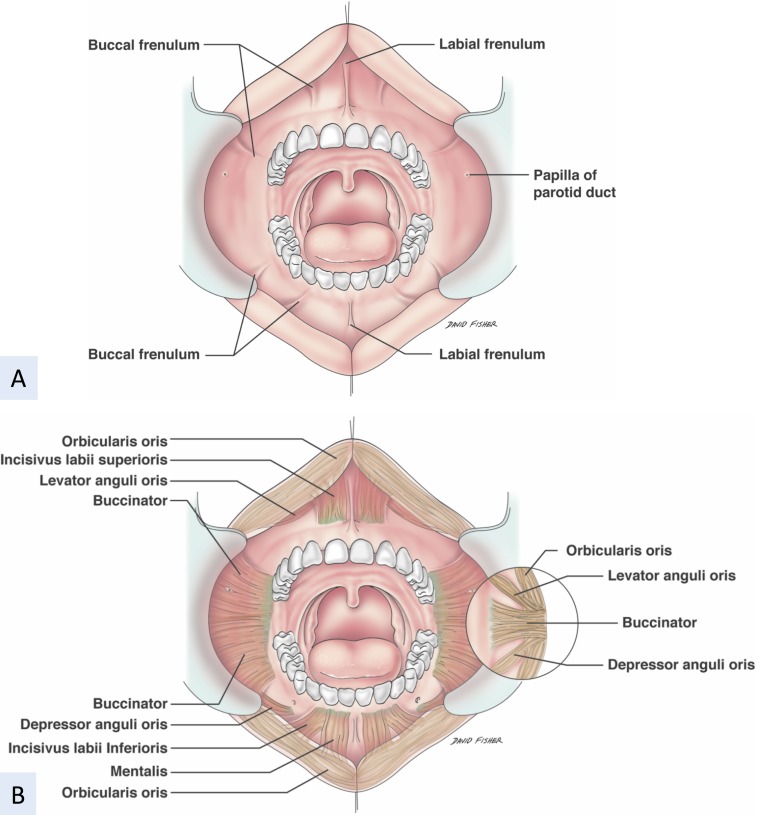
Schematic drawing of the mimetic muscles A: before dissection B: after removing the mucosa

The ILS muscle, buccinator, upper portion of the MT, and ILI lie under the oral mucosa (Figure 2).

Labial frenulum and mimetic muscles

The upper and lower labial frenula are located in the middle of the upper and lower parts of the oral vestibule, respectively. According to Gartner and Schein [11], a histological study of the labial frenulum demonstrated that muscle fibers and epithelial tissues exist deep to the labial frenulum, and they speculated those muscle fibers could be the cause of the unsuccessful outcome of the frenulectomy. Macroscopically, there is only taut connective tissue behind the labial frenulum [7]. The ILS is lateral to the connective tissue behind the upper frenulum and the MT lies lateral to the connective tissue behind the lower frenulum. The frenulum can be formed solely by taut connective tissue and not muscle. Some ILS fibers can be included in tissue specimens, especially if the two sides of the medial part of the ILS are close together.

Buccal frenulum and mimetic muscles

The buccal frenulum of the maxilla corresponds to the lateral border of the lower portion of the ILS and the anterior border of the buccinator. The buccal frenulum of the mandible corresponds to the lateral border of the upper portion of the MT and incisivus labii inferioris (ILI), and the anterior border of the buccinator [7]. In terms of periodontology, the buccal frenulum is considered one cause of gingival recession, and buccal frenulectomy of the high-positioned buccal frenulum might be indicated [12]. The easiest method is just to perform a frenulectomy. However, if the bony attachment of the muscles is not detached, the frenulum could retether during healing. Therefore, there is room for the development of new or modified frenulectomy procedures.

Mucogingival junction and mimetic muscles

Regarding the bony attachments of the mimetic muscles, the ILS, buccinator, MT, and ILI correspond to the mucogingival junction [5,7-8]. Consequently, the alveolar mucosa in the oral vestibule is basically supported by one of those mimetic muscles. Numerous periodontal surgeries have been developed and applied to the keratinized gingiva and alveolar mucosa without recognition that the mucogingival junction corresponds to the junction of the bone and the bony attachment of the mimetic muscles.

**Figure 3:**
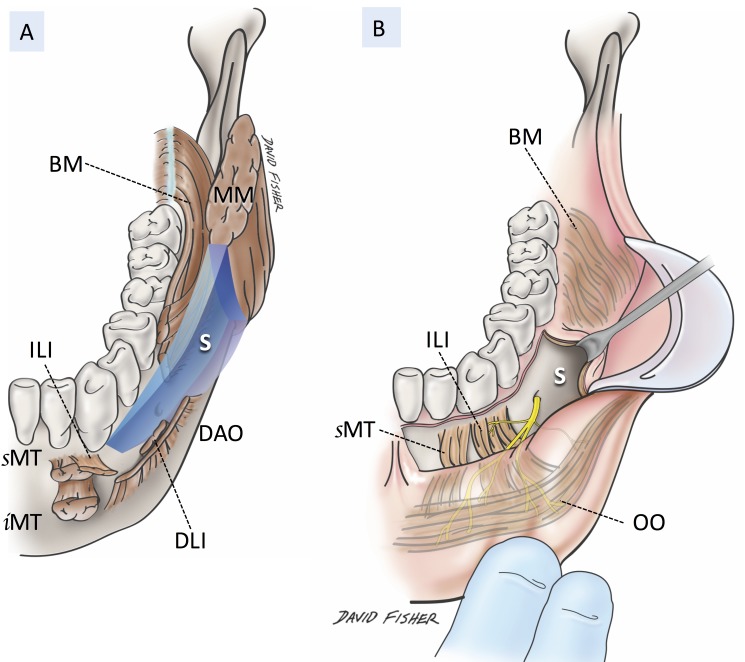
Schematic drawing of the buccomandibular space A: boundary of the buccomandibular space B: intraoral observation of the buccomandibular space BM; buccinator, DAO; depressor anguli oris, DLI; depressor labii inferioris, ILI; incisivus labii inferioris, MM; masseter muscle, MT; (inferior and superior portion of the) mentalis OO; orbicularis oris, PM; platysma, S; buccomandibular space

There are fissures between the mimetic muscles on both the maxilla and mandible. The fissure between the ILS and buccinators on the maxilla continues to the buccal space, and that between the MT and ILI and the buccinator on the mandible continues to the buccomandibular space (Figure 3) [6].

 Interestingly, this buccomandibular space has no continuity with the buccal space.

Other surgical procedures

Genioplasty of the mandible requires a transverse incision through the lower alveolar mucosa, and the upper portion of the MT has to be incised [13-14]. Le Fort surgery [15] for the maxilla requires a transverse incision into the upper alveolar mucosa, and the lower portion of the ILS has to be incised. However, the postoperative complications of this incision have seldom been discussed.

## Conclusions

A better understanding of the mimetic muscles from an intraoral perspective is important for those performing oral surgery and dentistry. With such knowledge, intraoperative and postoperative complications can be minimized. Additionally, with such a new perspective, the potential for new innovative procedures might be realized.

## References

[1] Standring S (2015). Gray's anatomy e-book: the anatomical basis of clinical practice. https://elsevier.ca/product.jsp?isbn=9780702052309.

[2] Watanabe K, Shoja MM, Loukas M, Tubbs RS (2015). Anatomy for plastic surgery of the face, head, and neck. Neck.

[3] Derby BM, Codner MA (2017). Evidence-based medicine: face lift. Plast Reconstr Surg.

[4] Mulliken JB, Wu JK, Padwa BL (2003). Repair of bilateral cleft lip: review, revisions, and reflections. J Craniofac Surg.

[5] Iwanaga J, Watanabe K, Schmidt CK (2017). Anatomical study and comprehensive review of the incisivus labii superioris muscle: application to lip and cosmetic surgery. Cureus.

[6] Iwanaga J, Kamura Y, Tsuyoshi T, Watanabe K, Kusukawa J, Oskouian RJ, Tubbs RS (2017). A new space of the face: the bucco-mandibular space. Clin Anat.

[7] Iwanaga J, Takeuchi N, Oskouian RJ, Tubbs RS (201). Clinical anatomy of the frenulum of the oral vestibule. Cureus.

[8] Iwanaga J, He P, Watanabe K, Kamura Y, Oskouian RJ, Tubbs RS (2017). Intraoral observation of the mentalis and incisivus labii inferioris muscles. J Craniofac Surg.

[9] Dubrul E (1988). Sicher and Dubrul's oral anatomy. https://books.google.com/books?id=hq5pAAAAMAAJ&source=gbs_book_other_versions.

[10] Hur MS, Kim HJ, Choi BY, Hu KS, Kim HJ, Lee KS (2013). Morphology of the mentalis muscle and its relationship with the orbicularis oris and incisivus labii inferioris muscles. J Craniofac Surg.

[11] Gartner LP, Schein D (1991). The superior labial frenum: a histologic observation. Quintessence Int.

[12] Toker H, Ozdemir H (2009). Gingival recession: epidemiology and risk indicators in a university dental hospital in Turkey. Int J Dent Hyg.

[13] Trainer R, Obwegeser H (1957). The surgical correction of mandibular prognathism and retrognathia with consideration of genioplasty. Part I. Surgical procedures to correct mandibular prognathism and reshaping of the chin. Oral Surg Oral Med Oral Pathol.

[14] Trainer R, Obwegeser H. (1957). The surgical correction of mandibular prognathism and retrognathia with consideration of genioplasty. Part II. Operating methods for microgenia and distoclusion. Oral Surg Oral Med Oral Pathol.

[15] Apinhasmit W (2005). Clinical anatomy of the posterior maxilla pertaining to Le Fort I osteotomy in Thais. Clin Anat.

